# Early Recurrence in Completely Resected IIIB and IIIC Melanoma Warrants Restaging Prior to Adjuvant Therapy

**DOI:** 10.1245/s10434-019-07274-2

**Published:** 2019-03-04

**Authors:** Martine Bloemendal, Wouter W. van Willigen, Kalijn F. Bol, Marye J. Boers-Sonderen, Johannes J. Bonenkamp, J. E. M. Werner, Erik H. J. G. Aarntzen, Rutger H. T. Koornstra, Jan Willem B. de Groot, I. Jolanda M. de Vries, Jacobus J. M. van der Hoeven, Winald R. Gerritsen, Johannes H. W. de Wilt

**Affiliations:** 1 Department of Medical Oncology, Radboud University Medical Center, Nijmegen, The Netherlands; 2 Department of Tumor Immunology, Radboud Institute for Molecular Life Sciences, Nijmegen, The Netherlands; 3 Department of Surgery, Radboud University Medical Center, Nijmegen, The Netherlands; 4 Department of Radiology and Nuclear Medicine, Radboud University Medical Center, Nijmegen, The Netherlands; 5 Oncological Center Isala, Zwolle, The Netherlands

## Abstract

**Purpose:**

To evaluate the results of restaging completely resected stage IIIB/C melanoma prior to start of adjuvant therapy.

**Patients and Methods:**

One hundred twenty patients with stage IIIB or IIIC (AJCC 2009) melanoma who underwent complete surgical resection were screened for inclusion in our trial investigating adjuvant dendritic cell therapy (NCT02993315). All patients underwent imaging to exclude local relapse or metastasis before entering the trial. The frequency of recurrent disease within 12 weeks after resection and the method of detection were investigated.

**Results:**

Sixty-nine (58%) stage IIIB and 51 (43%) stage IIIC melanoma patients were screened. Median age was 54 (range 27–79) years. Twenty-two (18%) of 120 patients with completely resected stage IIIB/C melanoma had evidence of early recurrent disease, despite exclusion thereof by prior imaging. Median interval between resection and detection of relapse was 7.4 (range 4.3–10.7) weeks. Recurrence was asymptomatic in 17 (77%) patients, but metastasis was noticed by the patient or physician in 5 (23%). Eight patients with local relapse received local treatment with curative intent, and one was treated with systemic therapy. The remaining patients had distant metastasis, 1 of whom underwent resection of a solitary liver metastasis while 12 patients received systemic treatment.

**Conclusions:**

Patients with completely resected stage IIIB/C melanoma have high risk of early recurrence before start of adjuvant therapy. Restaging should be considered for high-risk melanoma patients before start of adjuvant therapy.

Treatment of stage III melanoma consists of complete resection with curative intent. However, the risk of recurrence afterwards is high, resulting in 5-year overall survival (OS) rates between 40 and 78%.^1^–^3^ Therapeutic options and prospects for patients with metastatic melanoma have changed considerably in recent years, especially with the introduction of immune checkpoint inhibitors and BRAF and MEK inhibitors.^4^–^10^ These drugs have been proven to significantly improve OS in metastatic melanoma and have also shown promising results in the adjuvant setting. Phase III trials investigating adjuvant systemic therapy with ipilimumab (anti-CTLA-4 antibody) and combined dabrafenib/trametinib (BRAF/MEK-inhibitor) showed improved OS compared with placebo.^11^,^12^ Adjuvant nivolumab and pembrolizumab (both anti-PD-1 antibodies) led to improved 12-month recurrence-free survival (RFS) rates when compared with ipilimumab and placebo, respectively.^13^,^14^ Data on OS are still awaited. These results led to approval of ipilimumab, pembrolizumab, nivolumab, and combined dabrafenib/trametinib as adjuvant therapy by the Food and Drug Administration (FDA). The European Medicines Agency (EMA) approved use of nivolumab and combined dabrafenib/trametinib in the adjuvant setting and received a positive advice from the Committee for Medicinal Products for Human Use (CMHP) for adjuvant use of pembrolizumab.^15^–^23^

After diagnosis of nodal metastasis in high-risk stage III melanoma, imaging techniques [e.g. computed tomography (CT) or ^18^F-fluorodeoxyglucose (^18^F-FDG) positron emission tomography (PET)] are used to exclude distant metastasis. In stage IIIB/C melanoma, most recurrences appear within the first 2 years after surgical resection.^1^ Despite this high risk, incorporation of imaging techniques in follow-up after resection differs widely between centers. No survival benefit of imaging during follow-up was demonstrated in a randomized trial, but this was carried out prior to the introduction of effective therapies for metastatic melanoma.^24^,^25^ In a clinical trial investigating adjuvant therapy, it is mandatory to exclude recurrent disease prior to inclusion, preventing metastatic melanoma patients from entering the adjuvant study.

We report herein imaging results for 120 stage IIIB and IIIC melanoma patients who underwent complete surgical resection within 12 weeks prior to inclusion in a placebo-controlled, randomized trial investigating adjuvant dendritic cell therapy (NCT02993315). Imaging with contrast-enhanced venous-phase CT (ceCT) or ^18^F-FDG PET/CT was performed to exclude recurrent disease within 6 weeks prior to inclusion.

## Patients and Methods

### Patients

After signing informed consent, patients were screened for eligibility in a placebo-controlled randomized trial (NCT02993315) investigating adjuvant dendritic cell vaccination. The protocol has been approved by the national review committee (Central Committee on Research Involving Human Subjects) and is in concordance with the Declaration of Helsinki and Good Clinical Practice. Eligible patients were adults with stage IIIB or IIIC [American Joint Committee on Cancer (AJCC) 7th edition]^2^ cutaneous melanoma within 12 weeks after complete radical lymph node dissection (RLND) and after recovery from the surgery. The protocol was amended after publication of the MSLT-II trial results, which showed no survival benefit of completion lymph node dissection after removal of microscopic metastasis with sentinel node biopsy (SNB) when compared with nodal surveillance.^26^ After amendment, patients with microscopic disease could be included after SNB and additional completion lymph node dissection was no longer required. Macrometastasis was defined as a palpable node or as a nonpalpable node of at least 15 mm in short axis on CT, a PET-positive node, or one or more foci of melanoma of at least 1 cm in diameter in the pathology report. Patients with completely resected in-transit and/or satellite metastasis, an unknown primary tumor, and (planned) adjuvant radiotherapy could be included. In addition, absence of distant metastasis had to be documented by ceCT of the chest, abdomen, and pelvis or whole-body ^18^F-FDG PET scan combined with CT (^18^F-FDG PET/CT) within 6 weeks before inclusion in our trial. In patients with head or neck melanoma, additional ceCT of the neck was obligatory. Imaging of the brain was performed in case of clinical suspicion of brain metastasis. Exclusion criteria included autoimmune disease (except for skin disease, hypothyroidism after autoimmune thyroiditis, and type 1 diabetes mellitus), a second malignancy in the last 5 years (except for adequately treated carcinoma in situ and basal or squamous cell carcinoma of the skin), concomitant use of oral or intravenous immunosuppressive drugs, and uncontrolled infectious disease.

## Methods

Within 6 weeks prior to the start of the study, imaging to exclude relapse was performed. Recurrence was considered symptomatic if suspected by symptoms and/or abnormalities during physical examination. Otherwise, recurrence was considered asymptomatic. Blood tests, including lactate dehydrogenase (LDH), were carried out within 4 weeks before inclusion. For baseline characteristics, a conglomerate of lymph nodes with at least four metastatic lymph nodes and presence of extracapsular extension was regarded as N3 disease. In case of a conglomerate, the diameter of lymph node involvement was counted as the diameter of the conglomerate.

## Results

### Patient Characteristics

Between November 2016 and July 2018, 120 patients were screened for eligibility. Baseline characteristics are presented in Table 1. Median age was 54 (range 27–79) years, and 76 (63%) of patients were male. Sixty-nine (58%) and 51 (43%) patients were diagnosed with stage IIIB and IIIC melanoma, respectively. Twenty-one (18%) patients had completely resected in-transit metastasis, and nine (8%) patients presented with nodal metastasis from an unknown primary tumor. Baseline characteristics of patients with and without recurrent disease during screening are presented in Table 1. No statistically significant differences between groups were present.

**Table 1:** Baseline characteristics

Characteristic	Total (*n* = 120)	No recurrent disease during screening (*n* = 98)	Recurrent disease during screening (*n* = 22)
Median (range) age (years)	54 (27–79)	55 (27–79)	51 (27–73)
Sex, *n* (%)			
Male	76 (63)	59 (60)	17 (77)
Female	44 (37)	39 (40)	5 (23)
Stage at screening (AJCC 7th edition), *n* (%)			
IIIB	69 (58)	58 (59)	11 (50)
IIIC	51 (43)	40 (41)	11 (50)
Breslow, *n* (%)^a^			
< 2 mm	49 (44)	42 (47)	7 (32)
2–4 mm	24 (22)	19 (21)	5 (23)
≥ 4 mm	36 (32)	27 (30)	9 (41)
Other^b^	2 (2)	1 (1)	1 (5)
Ulceration, *n* (%)^a^			
Yes	38 (32)	31 (32)	7 (32)
No	73 (61)	58 (59)	15 (68)
Histological type, *n* (%)^a^			
Superficial spreading melanoma	73 (66)	61 (69)	12 (55)
Nodular melanoma	26 (23)	20 (22)	6 (27)
Other	7 (6)	5 (6)	2 (9)
Missing	5 (5)	3 (3)	2 (9)
Primary site, *n* (%)			
Head/neck	17 (14)	13 (13)	4 (18)
Trunk	46 (38)	37 (38)	9 (41)
Upper extremity	13 (11)	12 (12)	1 (5)
Lower extremity	34 (28)	26 (27)	8 (36)
Genital	1 (1)	1 (1)	0 (0)
Unknown primary	9 (8)	9 (9)	0 (0)
Type of lymph node involvement, *n* (%)			
Microscopic	21 (18)	19 (19)	2 (9)
Macroscopic	99 (83)	79 (81)	20 (91)
Median (range) maximum diameter of lymph node metastasis (cm)	2.0 (0.01–7.5)	1.9 (0.01–7.5)	3.0 (0.25–7.0)
Number of metastatic lymph nodes, *n* (%)			
0	1 (1)	1 (1)	0 (0)
1	46 (38)	40 (41)	6 (27)
2–3	37 (31)	29 (30)	8 (36)
≥ 4	36 (30)	28 (29)	8 (36)
Site of nodal metastasis			
Neck	26 (22)	21 (21)	5 (23)
Axilla	51 (43)	46 (47)	5 (23)
Groin	42 (35)	30 (31)	12 (55)
Popliteal	1 (1)	1 (1)	0 (0)
Extracapsular extension, *n* (%)			
Yes	30 (25)	23 (23)	7 (32)
No	67 (56)	56 (57)	11 (50)
Missing	23 (19)	19 (19)	4 (18)
In-transit or (micro)satellite metastases, *n* (%)^c^			
Yes	21 (18)	17 (17)	4 (18)
No	99 (83)	81 (83)	18 (82)
*BRAF*, *n* (%)			
*BRAF* V600E/V600 K	78 (65)	65 (66)	13 (59)
Wild type	34 (28)	29 (30)	5 (23)
Other^d^	3 (3)	2 (2)	1 (5)
Missing	5 (4)	2 (2)	3 (14)

^a^Excluding nine patients with unknown primary tumor

^b^Primary melanoma diagnosed as melanocytic tumor of uncertain malignant potential (MELTUMP) in two patients, confirmed by revision

^c^Including locoregional recurrences

^d^One inactivating mutation, one p.Leu485Trp mutation, one p.Thr599Dup mutation

*AJCC* American Joint Committee on Cancer, *BRAF* B-Raf proto-oncogene, serine/threonine kinase

### Detection of Recurrent Disease

**Fig. 1:**
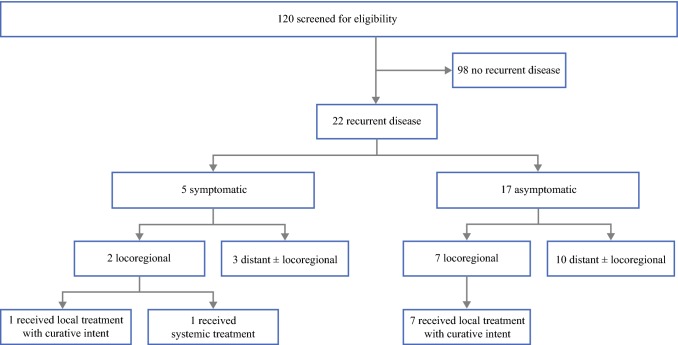
Detection of recurrent disease during screening for eligibility

Melanoma metastasis was detected in 22 (18%) of 120 patients (Fig. 1), corresponding to a number needed to screen of 5.45 to detect one patient with recurrent disease. Thirteen (59%) patients were identified with distant metastasis, while in the remaining nine (41%) patients, metastasis was locoregionally located.

Five (23%) recurrences were found based on symptoms or physical examination (symptomatic recurrence); in three patients, in-transit metastasis was noticed by the patient (*n* = 1) or physician (*n* = 2), and another patient discovered a local recurrence at the site of the resected primary melanoma. Of these four patients with symptomatic locoregional relapse, two showed detectable distant metastatic disease on ceCT scan. The fifth patient developed back pain, which was suspicious for bone metastasis and confirmed by ceCT imaging. Seventeen (77%) relapses were asymptomatic and initially detected by imaging, corresponding to a number of asymptomatic patients needed to screen of 6.76. One of these patients presented with atypical, very small pulmonary nodules before RLND. Another patient showed atypical/nonspecific hypodense liver lesions of maximum 10 mm on preoperative ceCT scan, and these lesions were identified as liver metastases during screening ceCT after a 12-week interval. Serum LDH level was not a sensitive parameter for recurrent disease, since only 4 (18%) out of 22 relapsed patients had elevated LDH. All four patients had distant metastasis, and two of them were asymptomatic.

### Imaging Techniques and Intervals

**Fig. 2:**
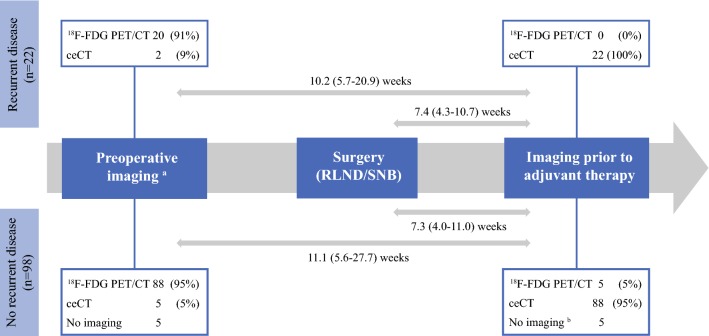
Time intervals and imaging techniques used prior to intended start of adjuvant therapy. Time intervals presented as median (range). ^a^Imaging prior to referral for trial participation was performed postoperatively after sentinel node biopsy (micrometastatic disease) in nine patients and in three patients with macrometastatic disease. ^b^Imaging was not repeated during screening for eligibility in five patients, since the inclusion in the adjuvant trial was within 6 weeks after prior imaging. *ceCT* contrast-enhanced venous phase CT; ^18^*F-FDG PET/CT*^18^F-fluorodeoxyglucose PET scan combined with CT, *RLND* radical lymph node dissection, *SNB* sentinel node biopsy

Before referral to our trial, metastasis had been excluded with ^18^F-FDG PET/CT (85%) or ceCT (15%) in 115 patients. Of the five patients in whom metastasis had not been excluded prior to screening, four had resected micrometastasis in the pathology report and one patient had macrometastatic disease. However, in all patients presenting recurrent disease during screening, distant metastasis had been excluded on imaging prior to start of screening for eligibility (Fig. 2). For this group with early relapse, prior imaging was done using ^18^F-FDG PET/CT in 20 patients (91%) and ceCT in the remaining 2 patients.

To screen for eligibility, 110 (96%) patients had standard ceCT. In the remaining five (4%) patients, imaging was performed by ^18^F-FDG PET/CT. Relapse during screening was detected by ceCT in all cases. In five patients, imaging was not repeated during screening, since the start of experimental adjuvant therapy was within 6 weeks after prior imaging excluding distant metastasis.

**Fig. 3:**
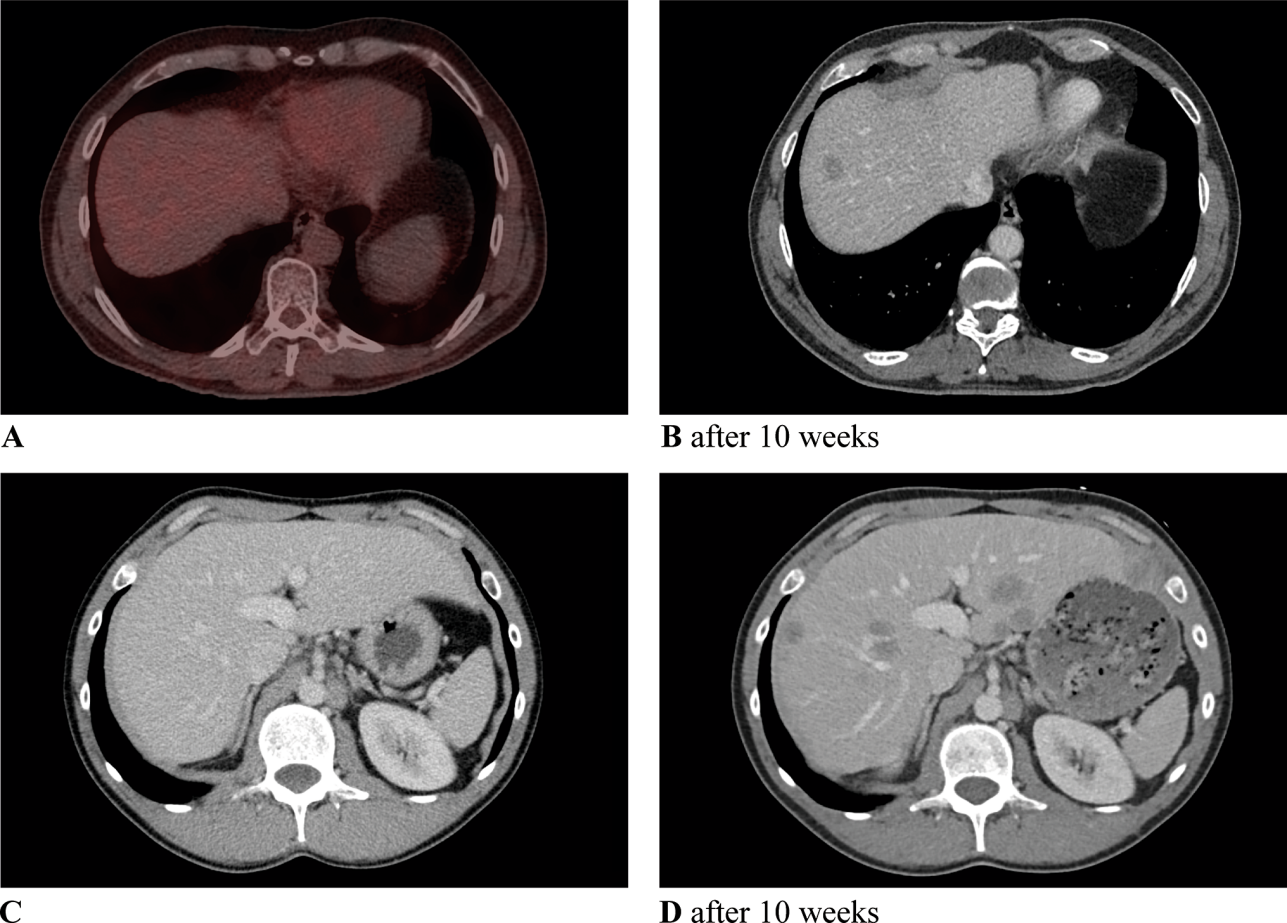
Asymptomatic recurrent melanoma during screening: A patient with pT4aN1b/stage IIIB melanoma (AJCC 7th edition)^2^ showed no metastatic disease on ^18^F-FDG PET/CT prior to radical lymph node dissection (RLND) (**a**), but venous-phase contrast-enhanced CT (ceCT) 10 weeks after RLND and 12 weeks after prior ^18^F-FDG PET/CT showed liver metastasis (**b**); A patient with pT2aN2b IIIB melanoma (AJCC 7th edition) showed no metastatic disease on ceCT (shown) and ^18^F-FDG PET/CT (not shown) prior to RLND (**c**), but ceCT 5 weeks after RLND and 10 weeks after prior ceCT revealed multiple liver metastases (**d**)

The median interval between imaging during screening and previous imaging was 10.2 (range 5.7–20.9) weeks in recurrent patients. The median interval between complete resection and detection of recurrent disease was 7.4 (range 4.3–10.7) weeks. In patients without recurrent disease, these intervals were not significantly different, with a median interval between scans of 11.1 (range 5.6–27.7) weeks and an interval between resection and imaging of 7.3 (range 4.0–11.0) weeks. Figure 3 shows examples of patients with asymptomatic recurrent disease.

### Treatment of Relapsed Patients

Nine patients showed locoregional metastasis, of whom eight were referred for surgical resection with curative intent. One patient had no evidence of disease after adjuvant radiotherapy, therefore planned surgery was cancelled. This patient was disease free during 13 months of follow-up, then relapsed. Of the seven reoperated patients, six developed recurrent disease. In two of them, distant metastasis occurred within 1 month after resection of the recurrent local disease. In four patients, the interval from resection to recurrent disease was 6, 6, 8, and 9 months. The last reoperated patient is still recurrence free after 10 months of follow-up. In the remaining patient, locoregional recurrence consisted of irresectable in-transit metastasis, for which treatment with anti-PD-1 antibodies was initiated.

Of the 13 patients with distant metastasis, first-line treatment consisted of anti-PD-1 antibodies in three patients, three patients started with combined immune checkpoint inhibition, and in six patients treatment with targeted therapy was initiated. One patient underwent metastasectomy of a solitary liver metastasis.

## Discussion

In 120 patients screened for an adjuvant trial, almost one out of five patients with completely resected stage IIIB or IIIC melanoma showed evidence of recurrent disease prior to start of adjuvant therapy, despite adequate prior imaging. These relapses were present within 2 months after surgery and within 3 months after previous staging. The majority of patients with recurrent disease were asymptomatic, and all were identified by ceCT scan.

Discovery of recurrent disease before start of adjuvant therapy improves information about prognosis. A proper baseline scan prevents incorrectly discarding therapy if metastasis is visualized at the first follow-up scan but was already present and detectable before start of therapy. In addition, evidence of relapse can change therapeutic management. About one-third of patients with recurrent disease were referred for additional resection with curative intent due to locoregional relapse. Furthermore, patients with rapid relapse with relatively high metastatic load started treatment with BRAF/MEK inhibitors or combined anti-CTLA-4/anti-PD-1 antibodies. Therefore, reimaging before start of adjuvant therapy leads to a change in therapeutic management in a substantial group of patients and should be considered in all patients despite prior imaging.

A limitation of this study is that we only evaluated patients screened for eligibility in our clinical trial, hence a selection bias might have occurred. Patients with more unfavorable prognosis and higher risk of recurrence are more likely to be referred for trial participation than patients who would be referred for approved adjuvant treatment. On the other hand, some rapid relapses are missed in our report due to development of symptomatic metastasis or due to recurrent disease diagnosed at radiotherapy planning CT scans before screening for eligibility. The interval between scans was similar between the groups with and without relapse, therefore a lead-time bias does not seem to play a role.

To the best of the authors’ knowledge, this is the first report about detection of early recurrent disease in resected stage III melanoma before start of adjuvant therapy. Studies have been conducted to analyze the discovery of metastasis by imaging in stage III melanoma patients during follow-up after resection.^27^–^34^ However, these studies performed imaging during follow-up with a longer interval after surgery and did not report recurrences in relation to start of adjuvant therapy. Mostly, the first scan was conducted 6–12 months after surgery, thus information about rapid asymptomatic relapses within 12 weeks is lacking. In line with our protocol, phase III trials investigating adjuvant treatment with anti-CTLA-4 or anti-PD-1 antibodies or BRAF/MEK inhibitors excluded metastasis with CT postoperatively and within 4–6 weeks prior to randomization.^11^–^14^ The trial investigating adjuvant ipilimumab versus nivolumab reported that 24% of screened resected stage IIIB/C/IV patients no longer met criteria and were not randomized.^13^ Exact numbers of screening failures due to recurrent disease were not mentioned but probably represent an important portion thereof. In addition, the contribution of relapse in stage IV melanoma patients, at higher risk for relapse than stage IIIB/C patients, is not reported. It would be interesting to analyze the numbers of recurrent disease during screening in the larger study cohorts of adjuvant phase III trials.

Taken together, about one-fifth of completely resected stage IIIB/C melanoma patients had recurrent disease before start of adjuvant treatment. Because of the impact on prognosis and therapeutic consequences, restaging all high-risk patients before start of adjuvant therapy seems appropriate.
